# Therapeutic Effects of Engineered Exosomes from RAW264.7 Cells Overexpressing hsa-let-7i-5p against Sepsis in Mice—A Comparative Study with Human Placenta-Derived Mesenchymal Stem Cell Exosomes

**DOI:** 10.3390/jpm14060619

**Published:** 2024-06-09

**Authors:** Van Long Le, Chao-Yuan Chang, Ching-Wei Chuang, Syuan-Hao Syu, Hung-Jen Shih, Hong-Phuc Nguyen Vo, Minh Nguyen Van, Chun-Jen Huang

**Affiliations:** 1 International Ph.D. Program in Medicine, College of Medicine, Taipei Medical University, Taipei 110, Taiwan; d142109014@tmu.edu.tw (V.L.L.); d142111007@tmu.edu.tw (H.-P.N.V.); 2 Department of Anesthesiology and Intensive Care & Emergency Medicine, Hue University of Medicine and Pharmacy, Hue City 52000, Vietnam; 3 Department of Medical Research, Wan Fang Hospital, Taipei Medical University, Taipei 116, Taiwan; 110234@w.tmu.edu.tw; 4 Integrative Research Center for Critical Care, Wan Fang Hospital, Taipei Medical University, Taipei 116, Taiwan; 111044@w.tmu.edu.tw (C.-W.C.); 100454@w.tmu.edu.tw (S.-H.S.); 5 Graduate Institute of Clinical Medicine, College of Medicine, Taipei Medical University, Taipei 110, Taiwan; 6 Department of Anesthesiology, Wan Fang Hospital, Taipei Medical University, Taipei 116, Taiwan; 7 Department of Anesthesiology, School of Medicine, College of Medicine, Taipei Medical University, Taipei 110, Taiwan; 8 Department of Urology, School of Medicine, College of Medicine, Taipei Medical University, Taipei 110, Taiwan; 9 Department of Urology, Wan Fang Hospital, Taipei Medical University, Taipei 116, Taiwan; 10 Division of Urology, Department of Surgery, Changhua Christian Hospital, Changhua 500, Taiwan; jasta1206@gmail.com; 11 Department of Post-Baccalaureate Medicine, College of Medicine, National Chung Hsing University, Taichung 402, Taiwan; 12 Department of Anesthesiology, College of Medicine, Can Tho University of Medicine and Pharmacy, Can Tho City 900000, Vietnam

**Keywords:** exosomes, hsa-let-7i-5p, Raw264.7 cells, stem cells, lung, endotoxin, mice

## Abstract

This study compared the therapeutic effects of engineered exosomes derived from RAW264.7 cells overexpressing hsa-let-7i-5p (engineered exosomes) to exosomes from human placenta-derived mesenchymal stem cells (hpMSC exosomes) against sepsis-induced acute lung injury. Adult male C57BL/6 mice were divided into lipopolysaccharide (LPS), LPS plus engineered exosome (LEExo), or LPS plus hpMSC exosome (LMExo) groups, alongside control groups. The results showed that lung injury scores (based on pathohistological characteristics) and the levels of lung function alterations, tissue edema, and leukocyte infiltration in LEExo and LMExo groups were comparable and significantly lower than in the LPS group (all *p* < 0.05). Furthermore, the levels of inflammation (nuclear factor-κB activation, cytokine upregulation), macrophage activation (hypoxia-inducible factor-1α activation, M1 phase polarization), oxidation, and apoptosis were diminished in LEExo and LMExo groups compared to the LPS group (all *p* < 0.05). Inhibition of hsa-let-7i-5p attenuated the therapeutic effects of both engineered and hpMSC exosomes. These findings underscore the potent therapeutic capacity of engineered exosomes enriched with hsa-let-7i-5p and their potential as an alternative to hpMSC exosomes for sepsis treatment. Continued research into the mechanisms of action and optimization of engineered exosomes could pave the way for their future clinical application.

## 1. Introduction

Sepsis stands as a critical medical challenge marked by an aberrant immune response to infection, presenting a substantial danger to life [1,2]. This syndrome manifests as uncontrolled inflammation, often resulting in multiorgan dysfunction, notably affecting the lungs [1,2]. According to Rudd et al., in 2017 alone, there were approximately 50 million reported cases of sepsis worldwide, with over 10 million deaths attributed to the condition, representing nearly 20% of all global fatalities [3]. Despite its staggering impact, effective treatments for sepsis are still elusive [3], underscoring an urgent demand for innovative therapeutic intervention.

At the center of sepsis pathophysiology lies complex mechanisms involving inflammation, oxidative stress, and apoptosis [4,5]. Activation of pattern-recognition receptors triggers the release of pro-inflammatory cytokines and inflammasome activation, exacerbating the condition [6]. Additionally, disruptions in the antioxidant system exacerbate endothelial dysfunction and systemic inflammation [7]. Notably, heightened inflammation and oxidative stress synergistically contribute to the induction of programmed cell death, namely apoptosis [4,5,6,7]. Consequently, interventions targeting robust inflammation and oxidative stress mechanisms may potentially mitigate apoptosis and yield beneficial effects against sepsis [8,9,10].

Mesenchymal stem cells (MSCs), identified in the 1970s, have garnered attention for their immunomodulatory and antioxidative properties [11,12,13]. Particularly intriguing are their extracellular vesicles, notably exosomes, which hold promise in therapeutic applications [14,15]. Ranging from 50 to –200 nm, these nanosized vesicles offer advantages in accessibility and manufacturability compared to MSCs themselves [16]. Much like MSCs, MSC-derived exosomes (MSC exosomes) demonstrate immunomodulatory and antioxidative effects, influencing various immune cells and cytokines pivotal in sepsis pathogenesis [14,15]. Noteworthy, studies have highlighted the therapeutic potential of MSC exosomes in alleviating lung injury associated with sepsis [17,18].

One crucial mechanism through which MSC exosomes exert therapeutic effects is by delivering therapeutic cargo, such as microRNAs (miRNAs) [19]. MicroRNAs, pivotal regulators of gene expression, play essential roles in modulating the immune response during sepsis [19,20]. In our previous investigation, we identified hsa-let-7i-5p as one of the most abundant miRNAs in human placenta-derived MSC (hpMSC) exosomes [18,21]. Previous data have indicated that hsa-let-7i-5p possesses potent anti-inflammatory and antioxidative properties [18,21,22]. In a cellular model of myocardial ischemia, the overexpression of let-7i-5p was found to suppress hypoxia-induced NF-κB activation, mitochondrial dysfunction, and apoptosis [22]. Furthermore, our findings demonstrated that inhibition of hsa-let-7i-5p abolished the therapeutic effects of hpMSC exosomes against sepsis in obese mice [18,21], underscoring the crucial role of hsa-let-7i-5p in mediating the therapeutic effects of hpMSC exosomes in sepsis.

One of the primary limitations of MSC exosomes in clinical practice arises from the restricted availability of MSC sources [23]. A promising strategy involves generating engineered exosomes from non-stem cell sources loaded with specific miRNAs, such as let-7i-5p, to offer therapeutic effects akin to those of MSC exosomes. To delve deeper into this approach, we produced engineered exosomes utilizing RAW264.7 cells (an immortalized murine macrophage cell line) overexpressing hsa-let-7i-5p mediated by plasmid delivery. Subsequently, we investigated the therapeutic potential of these engineered exosomes against sepsis, hypothesizing that engineered exosomes enriched with hsa-let-7i-5p could improve survival rates and mitigate acute lung injury in lipopolysaccharide (LPS)-induced monomicrobial sepsis mice. Furthermore, since the objective of this study was to identify a novel therapy that could potentially serve as a viable alternative to MSC exosomes for treating sepsis, the therapeutic effectiveness of engineered exosomes enriched with hsa-let-7i-5p was compared to that of hpMSC exosomes in this study.

## 2. Materials and Methods

### 2.1. Cell Cultures and Animals

#### 2.1.1. Cell Cultures

This study employed RAW264.7 cells and hpMSCs to facilitate investigations. RAW264.7 cells were purchased from the Bioresource Collection and Research Center, Taiwan (Hsinchu, Taiwan). Human placenta-derived MSCs were provided by Professor Yen-Huan Huang from Taipei Medical University, Taipei, Taiwan (N202101014: approval from the Joint Institutional Review Board of Taipei Medical University regarding the human study of human placenta acquisition and hpMSC isolation). RAW264.7 cells and hpMSCs were maintained as previously reported [18,21,24].

#### 2.1.2. Animals

The Institutional Animal Use and Care Committee of Taipei Medical University approved all animal studies (LAC-2020-0373). Adult male wild-type C57B/L6 mice (7–8 weeks old, Taiwan National Laboratory Animal Center, Taipei, Taiwan) were used for experiments. Regular laboratory chow and water were provided to all mice with free access. Mice were maintained on a 12:12 h light–dark cycle. Care and handling of the mice were performed following the US National Institutes of Health guidelines.

### 2.2. Genetic Modification of RAW264.7 Cells for Overexpressing hsa-let-7i-5p

Confluent RAW264.7 cells were cultured in Dulbecco’s modified Eagle’s medium (DMEM; Life Technologies, Carlsbad, CA, USA), supplemented with 10% fetal bovine serum and 1% penicillin–streptomycin (all from Life Technologies), and maintained in a humidified incubator with 5% CO_2_ in air. After adherence, the RAW264.7 cells were cultured in serum-free medium (Life Technologies) for 30 min, followed by transfection with plasmids (2 μg) overexpressing hsa-let-7i-5p (pCMV-let-7i-5p, SC400011; OriGene Technologies, Rockville, MD, USA). After reaction for 5 min, serum-free medium (205 µL) containing Lipofectamine 3000 (5 µL) (both from Thermo Fisher, Waltham, MA, USA) was added to the mixtures. The hsa-let-7i-5p-overexpressing stable cell lines were then screened with neomycin.

### 2.3. Isolation of Engineered Exosomes and hpMSC Exosomes

Isolation of engineered exosomes from RAW264.7 cells overexpressing hsa-let-7i-5p (engineered exosomes) and exosomes from hpMSC (hpMSC exosomes) were performed as previously reported [18,21]. In brief, culture medium was harvested and centrifuged (Beckman Coulter Allegra X-15R centrifuge, 300× *g*, 4 °C, 10 min). Supernatants were filtered (0.22 mm filters, Millipore, Burlington, MA, USA) and then ultracentrifuged (Beckman Coulter OptimaTM L-80XP Ultracentrifuge, 100,000× *g*, 4 °C, 90 min, with a Type 50.2 Ti rotor, k-factor: 157.7) to pellet exosomes. The pellets were resuspended and then pooled, ultracentrifuged, resuspended, purified, and again ultracentrifuged. The top fractions of the gradient were then collected, diluted, and centrifuged. The crude exosome-containing pellets were resuspended in 1 mL of ice-cold PBS and pooled. A second round of ultracentrifugation was performed, and the resulting exosome pellets were resuspended again in 500 μL of phosphate-buffered saline (PBS, Life Technologies) and stored (−80 °C).

### 2.4. Confirmation of Isolated Exosomes and miRNA Analysis in Engineered Exosomes

#### 2.4.1. Morphology

Morphology of the isolated engineered exosomes and hpMSC exosomes was confirmed using transmission electron microscopy (TEM), as previously reported [18,21]. For TEM, exosome suspensions (3 μL) were fixed (50 μL 2% paraformaldehyde, Sigma-Aldrich, Saint Louise, MO, USA) and then transferred onto 2 Formvar-carbon-coated electron microscopy grids, followed by observation with a transmission electron microscope (JEM 1400 series, JEOL USA, Peabody, MA, USA).

#### 2.4.2. Particle Sizing Analysis

For exosome particle sizing analysis, the NanoSight NS300 instrument (Nanosight, Malvern Panalytical, Malvern, UK) was used, as previously reported [18,21]. Analysis was performed according to the manufacturer’s protocols.

#### 2.4.3. Markers of Exosomes

For the analysis of exosome markers ALIX and CD9 [25], a capillary-based Western blot analysis (the Simple Western method) was conducted using the WES system (ProteinSimple, Santa Clara, CA, USA) [26]. Protein samples were diluted and prepared in accordance with the manufacturer’s protocol. After denaturation (95 °C, 5 min), the samples were loaded onto the plate. Electrophoretic separation, antibody incubation, and chemiluminescence detection were performed within the WES system using default settings. Primary antibodies targeting ALIX (anti-ALIX antibody, ab235377; Abcam, Cambridge, UK) and CD9 (anti-CD9 antibody, IR300-981; iReal Technology, Hsinchu, Taiwan) were utilized. The digital image was analyzed using Compass software (Compass for SW, Version 6.3.0; ProteinSimple), with quantified data of the detected proteins reported in terms of molecular weight and signal/peak intensity.

#### 2.4.4. Analysis of hsa-let-7i-5p in Engineered Exosomes

To analyze the miRNA hsa-let-7i-5p levels in exosomes, droplet digital PCR (ddPCR) was utilized. Total RNA was extracted from hpMSC exosomes and engineered exosomes using the miRNeasy Serum/Plasma Kit (Qiagen, Hilden, Germany) following the recommended procedure. Reverse transcription was conducted with the TaqMan^®^ MicroRNA Assay (Thermo Fisher) as per the manufacturer’s instructions. The miRNA copy numbers were then analyzed using the QX200 ddPCR system (Bio-Rad, Hercules, CA, USA). In accordance with the manufacturer’s protocol, the reverse transcription products, primers, master mix, and mineral oil were loaded into a droplet generator to generate thousands of droplets. PCR amplification was carried out with the TaqMan^®^ MicroRNA Assay (Thermo Fisher). Subsequently, the droplets were aspirated and read using the Droplet Reader (Bio-Rad). Data analysis was performed using QUANTASOFT analysis software (Version 1.7; Bio-Rad). Technical support for this study was provided by the National Genomics Center for Clinical and Biotechnological Applications, National Yang-Ming Chiao Tung University, Taipei, Taiwan.

### 2.5. Electroporation of hsa-let-7i-5p Inhibitor into Exosomes

The role of let-7i-5p miRNA was further elucidated by employing a let-7i-5p miRNA inhibitor (Biotools, New Taipei City, Taiwan), as we previously reported [18,21]. The sequence used for let-7i-5p inhibition was 5′-AACAGCACAAACUACUACCUCA-3′. Electroporation of the miRNA inhibitor into engineered exosomes and MSC exosomes was performed through precipitation, resuspension, transferal, electroporation (150 V/100 μF), removal of free-floating miRNA, and ultracentrifugation [18]. The pellets of miRNA inhibitor-treated engineered exosomes and MSC exosomes were resuspended and stored at −80 °C.

### 2.6. Biodistribution and Pharmacokinetics of Exosomes

#### 2.6.1. Biodistribution Assay

Biodistribution assay was performed, as previously reported [18,21]. An independent cohort of 24 mice was used for this assay. Divided into two groups (*n* = 12 in each group), the first group of mice received intraperitoneal injections of hpMSC exosomes labeled with Cy7 mono NHS ester (Amersham Biosciences, Buckinghamshire, UK) at a dose of 1 × 10^8^ particles per mouse. The second group of mice received engineered exosomes, also labeled with Cy7 mono NHS ester (Amersham), at a dose of 1 × 10^9^ particles per mouse. Three mice from each group were sacrificed at 0, 2, 24, and 48 h via decapitation, and all vital organs were collected. The Cy7 signal was detected using a bioluminescence imaging assay (IVIS Lumina XRMS and Living Image software, Version 4.7.3; PerkinElmer, Shelton, CT, USA).

#### 2.6.2. Pharmacokinetics

An assay of pharmacokinetics was performed, also as previously reported [18,21]. Another independent cohort of 6 mice was used for this assay. Also divided into two groups (*n* = 3 in each group), the first group of mice received intraperitoneal injections of hpMSC exosomes labeled with Cy7 mono NHS ester (Amersham) (1 × 10^8^ particles per mouse), and the second group of mice received engineered exosomes, also labeled with Cy7 mono NHS ester (Amersham) (1 × 10^9^ particles per mouse). Serial blood sampling was performed in the 3 mice from each group, through submandibular vein puncture before administration (0 h) and at 2, 4, 24, and 48 h after administration. The fluorescence of each sample was measured using the SpectraMax M5 microplate reader (Molecular Devices, San Jose, CA, USA), with excitation at 756 nm and an emission peak at 779 nm, to facilitate pharmacokinetic analysis of hpMSC exosomes and engineered exosomes, respectively.

### 2.7. Therapeutic Effects of Exosomes against Sepsis

#### 2.7.1. Monomicrobial Sepsis Model

As mentioned above, this study employed a widely used LPS-induced monomicrobial sepsis model to facilitate the investigation. In brief, intraperitoneal injection of gram (-) endotoxin (25 mg/kg, LPS, *Escherichia coli* 0127:B8, Sigma-Aldrich) was performed as previously reported [27].

#### 2.7.2. Experimental Protocols

To facilitate investigations, the mice were randomized into different groups and subjected to various treatments, including intraperitoneal injection of normal saline (0.5 mL, the Sham group); normal saline supplemented with hpMSC exosomes (the MExo group); normal saline supplemented with engineered exosomes (the EExo group); LPS alone (the LPS group); LPS plus hpMSC exosomes (the LMExo group); LPS plus engineered exosomes (the LEExo group); LPS plus inhibitor-treated hpMSC exosomes (the LMExoi group); or LPS plus inhibitor-treated engineered exosomes (the LEExoi group).

In the MExo group and LMExo group, two doses of hpMSC exosomes (1 × 10^8^ particles per mouse) were administered intraperitoneally at 2 and 26 h after normal saline or LPS injection, respectively. In the EExo and LEExo groups, two doses of engineered exosomes (1 × 10^9^ particles per mouse) were administered intraperitoneally at 2 and 26 h after normal saline or LPS injection, respectively. Similarly, in the LMExoi group and LEExoi group, two doses of inhibitor-treated hpMSC exosomes (1 × 10^8^ particles per mouse) and inhibitor-treated engineered exosomes (1 × 10^9^ particles per mouse), respectively, were administered intraperitoneally at 2 and 26 h after LPS injection.

The dosages of MSC exosomes and inhibitor-treated MSC exosomes were determined based on our previous data obtained from obese mice complicated with sepsis [18,21]. Additionally, the dosage of engineered exosomes was determined based on our preliminary data (please refer to the Supplemental Figure S1), indicating that two doses of 1 × 10^9^ particles per mouse, but not 1 × 10^8^ particles per mouse, significantly improved survivorship in LPS-treated mice. Consequently, the dosage of inhibitor-treated engineered exosomes was determined accordingly.

#### 2.7.3. Survivorship, Plasma Sample Collection, and Plasma Cytokine Measurements

An independent cohort of 78 mice was used for this assay. Among them, 18 mice were allocated to the Sham, MExo, and EExo groups (*n* = 6 in each group) and 60 of them were allocated to the LPS, LMExo, LMexoi, LEExo, and LEExoi groups (*n* = 12 in each group). All mice were closely monitored for 48 h to determine the 48-h survival rate in each group, with the death recognized as blood pressure pulsation disappearance. The survivorship differences between groups were compared using the Kaplan–Meier survivorship plots.

After the completion of the survivorship determination, 5 surviving mice from each group were anesthetized, and blood samples were obtained via cardiac puncture to facilitate investigation of systemic inflammation levels. This was assessed by measuring cytokine levels in collected plasma samples. These blood samples were then placed into heparin tubes (Venosafe; Terumo Europe, Leuven, Belgium) and centrifuged at 2000× *g* for 10 min. The resulting supernatant plasma samples were collected and stored at −20 °C for subsequent analysis.

The plasma concentrations of cytokines were determined within 7 days after sample collection, using enzyme-linked immunosorbent assay (ELISA). The levels of cytokines, including tumor necrosis factor-α (TNF-α), interleukin-1β (IL-1β), and IL-6, in plasma were assessed using ELISA kits for TNF-α, IL-1β, and IL-6 (all from Enzo Life Science, Farmingdale, NY, USA), and performed as per the manufacturer’s protocols.

#### 2.7.4. Functional Assay of the Lungs

An independent cohort of 80 mice was used for this assay. Among them, 18 mice were allocated to the Sham, MExo, and EExo groups (*n* = 6 in each group), 42 mice were allocated to the LPS, LMexoi, and LEExoi groups (*n* = 14 in each group), and 20 mice were allocated to the LMexo and LEExo groups (*n* = 10 in each group). The sample size of each group was determined based on the 48-h survival rate data, to ensure each group would have at least 6 surviving mice for this assay. At 48 h after LPS or saline injection, 6 surviving mice from each group were anesthetized (zoletil/xylazine, 40/10 mg/kg, i.p.). Subsequently, the mice underwent tracheostomy and insertion of a tracheostomy tube (22# intravenous catheter; Terumo Corp., Tokyo, Japan) to facilitate pulmonary function assay using a computerized small animal ventilator (flexiVent FX; SCIREQ Inc., Montreal, QC, Canada). The mechanical ventilation was set at a ventilation rate of 150 breaths/min and a tidal volume of 0.2 mL. Parameters, including inspiratory capacity, airway resistance, and dynamic compliance, were recorded using a flexiWare 8 System (SCIREQ) [18].

#### 2.7.5. Lung Tissue Collection and Bronchoalveolar Lavage Fluid (BALF) Collection

An independent cohort of 240 mice was used for the following assays. Among them, 54 mice were allocated to the Sham, MExo, and EExo groups (*n* = 18 in each group), 126 mice were allocated to the LPS, LMexoi, and LEExoi groups (*n* = 42 in each group), and 60 mice were allocated to the LMexo and LEExo groups (*n* = 30 in each group). The sample size of each group was determined based on the 48-h survival rate data, to ensure each group would have at least 18 surviving mice for the following assays.

At 48 h after LPS or saline injection, following euthanasia by decapitation, the lung tissues of the first subset of 6 surviving mice from each group were removed en bloc and promptly snap-frozen in liquid nitrogen, preserving them at −80 °C for subsequent analysis. For the second subset of 6 surviving mice from each group, following euthanasia by decapitation and tracheostomy with tracheostomy tube insertion, the left main bronchus of mice from each group was ligated. Then, the left lung lobes were freshly harvested for wet/dry weight ratio assays to assess lung edema levels [18]. Then, the right lungs were infused with 10% formaldehyde (Sigma-Aldrich) under constant pressure for staining purposes and then harvested.

In addition, the third subset of 6 surviving mice from each group received bronchoalveolar lavage with 1 mL aliquots of sterile normal saline 5 times, and the bronchoalveolar lavage fluid (BALF) was collected. The total cell counts and differential cell counts were then determined [18].

#### 2.7.6. Histological Analysis of Lung Injury and Wet/Dry Weight Ratio Assay

The formaldehyde-infused right lung tissues from the second set of 6 surviving mice in each group were embedded in paraffin wax, serial sectioned, and then stained with hematoxylin and eosin. Morphological characteristics of lung injury were evaluated under a light microscope based on the features of alveolar wall edema, vascular congestion, hemorrhage, and polymorphonuclear (PMN) leukocyte infiltration [18]. Based on the lung injury score scale (0: normal, 5: severe), each histological characteristic was rated, and the sum was calculated to determine lung injury levels.

For the wet/dry weight ratio assay (namely the indicator of tissue water content), the freshly harvested left lung tissues, also from the second set of 6 surviving mice in each group, were weighed and then placed in the oven (80 °C, 24 h) and weighed again when dry. The wet/dry weight ratio was then determined.

#### 2.7.7. Lung Inflammation Assay

a. - Activation of upstream regulator nuclear factor-κB (NF-κB)

The snap-frozen lung tissues from 5 mice, randomly selected from the first set of 6 surviving mice in each group, were used for this assay. Activation of upstream regulator NF-κB in lung tissues was assayed using the Simple Western method, which was conducted as described above. Primary antibodies targeting phosphorylated NF-κB (3033L, Cell Signaling Technology, Danvers, MA, USA) and Actin (A2228, Sigma-Aldrich) were employed.

b. Cytokines in the lungs

The pulmonary concentrations of cytokines were also determined using ELISA. The snap-frozen lung tissues from 5 mice, randomly selected from the first set of 6 surviving mice in each group, were used for this assay. Lung tissue processing followed previously established protocols [18]. The levels of cytokines, including TNF-α, IL-1β, and IL-6, in lung tissues were assessed using ELISA kits for TNF-α, IL-1β, and IL-6 (all from Enzo Life Science).

c. Macrophage M1 phase polarization

The snap-frozen lung tissues from 5 mice, randomly selected from the first set of 6 surviving mice in each group, were used for this assay. Activation of hypoxia-inducible factor-1α (HIF-1α, macrophage M1 phase polarization promoter) and inducible nitric oxide synthase (iNOS, macrophage M1 phase polarization marker) in lung tissues was assayed also using the Simple Western method, which was conducted as described above. Primary antibodies targeting HIF-1α (IR113-466, iReal Technology), iNOS (IR231-856, iReal Technology), and Actin (A2228, Sigma-Aldrich) were used.

Expression of iNOS was also assayed using immunohistochemistry staining of iNOS in paraffin sections of lung tissues [18]. The formaldehyde-infused left lung tissues from 5 mice, randomly selected from the second set of 6 surviving mice in each group, were used for this assay. Tissue sections were processed and then incubated with anti-iNOS antibodies (IR231-856, iReal Technology), followed by scanning (TissueGnostics Axio Observer Z1 microscope; TissueGnostics GmbH, Austria) and analysis (Image J, Version 1.52e; free software by NIH, USA; available at https://imagej.nih.gov/ij/; last accessed date: 1 August 2021).

#### 2.7.8. Lung Oxidation Assay

a. Assay of endogenous antioxidant enzymes

The snap-frozen lung tissues from 5 mice, randomly selected from the first set of 6 surviving mice in each group, were used for this assay. Expression of oxidation regulatory enzyme superoxide dismutase 2 (SOD-2) was also measured using the Simple Western method, which was conducted as described above. The primary antibodies targeting SOD-2 (ab68155, Abcam, Cambridge, MA, USA) and Actin (A2228, Sigma-Aldrich) were used.

b. Assay of lipid peroxidation

Expression of the marker of oxidative stress was assayed using immunohistochemistry staining of lipid peroxidation-related protein malondialdehyde (MDA, ab27642, Abcam) [18]. The formaldehyde-infused left lung tissues from 5 mice, randomly selected from the second set of 6 surviving mice in each group, were used for this assay. After processing and incubaion with MDA, all tissue sections were observed (TissueGnostics Axio Observer Z1 microscope, TissueGnostics) and analyzed (Image J, provide in Section 2.7.7 c.).

#### 2.7.9. Cell Death Process Apoptosis in the Lungs

a. Assay of pro-apoptotic protein

The snap-frozen lung tissues from 5 mice, randomly selected from the first set of 6 surviving mice in each group, were used for this assay. Expression of the pro-apoptotic protein cleaved caspase-3 was also assayed using the Simple Western method, which was conducted as described above. The primary antibodies targeting cleaved caspase-3 (IR96-401, iReal Technology) and Actin (A2228, Sigma-Aldrich) were employed.

b. Terminal deoxynucleotidyl transferase dUTP nick end labeling (TUNEL)

Apoptosis was measured using the TUNEL method to detect apoptotic cells in lung tissues [18] using an in-situ cell death detection kit (Roche, Basel, Switzerland). The formaldehyde-infused left lung tissues from 5 mice, randomly selected from the second set of 6 surviving mice in each group, were used for this assay. After staining with DAPI (Pierce, Appleton, WI, USA) to detect total nuclei, tissue sections were visualized using a confocal microscope (LMS-780; Zeiss, Oberkochen, Germany), and the TUNEL positive ratio was calculated, as we previously reported [18].

### 2.8. Statistical Analysis

Data were presented as mean ± standard deviations. Between-group differences were analyzed using one-way analysis of variance and post hoc pairwise comparisons with Tukey’s test. The Kaplan–Meier analysis was performed to analyze the 48-h survival rates. A *p*-value of <0.05 was considered statistically significant.

## 3. Results

### 3.1. Confirmation of Exosomes

**Figure 1:**
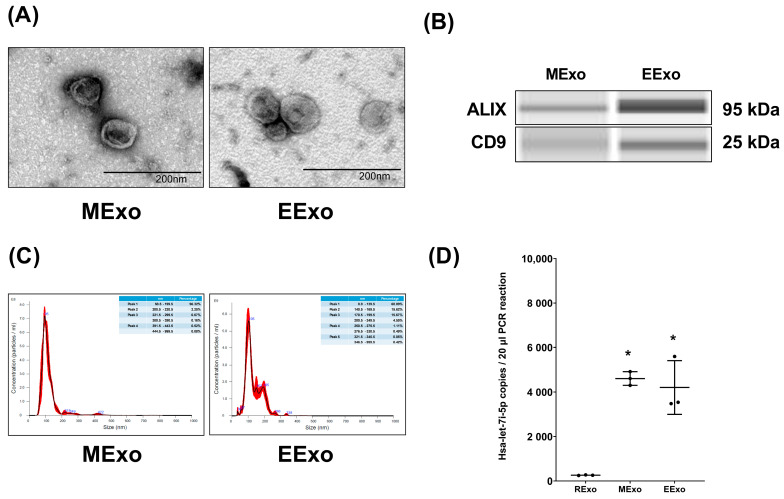
Characteristics of isolated exosomes. (**A**) Representative transmission electron microscopic images of isolated exosomes. (**B**) Representative gel photography of markers ALIX and CD63 in isolated exosomes using the Simple Western method. (**C**) Particle sizing assay of isolated exosomes. (**D**) The microRNA concentrations of hsa-let-7i-5p in isolated exosomes. Data were obtained from 3 exosomes in each group and presented as mean ± standard deviations. * *p* < 0.05, versus the RExo group. EExo: engineered exosomes from RAW264.7 cells overexpressing hsa-let-7i-5p. MExo: exosomes from human placenta-derived mesenchymal stem cells. RExo: exosomes from RAW264.7 cells without genetic modification.

Both hpMSC exosomes and engineered exosomes exhibited a characteristic double-layered cup-shaped morphology under TEM, as depicted in Figure 1A. Immunoblotting assays confirmed the presence of positive markers ALIX and CD63 (Figure 1B). Nanoparticle tracking analysis revealed particle sizes of approximately 50–200 nm for both types of exosomes (Figure 1C). Furthermore, our data indicated comparable miRNA concentrations of hsa-let-7i-5p in both hpMSC exosomes and engineered exosomes (4610 ± 310 and 4207 ± 1207 copies, respectively), and both were significantly higher than that in exosomes isolated from RAW264.7 cells without genetic modification (259 ± 14 copies) (both *p* < 0.001) (Figure 1D).

### 3.2. Biodistribution and Pharmacokinetics of Exosomes

**Figure 2:**
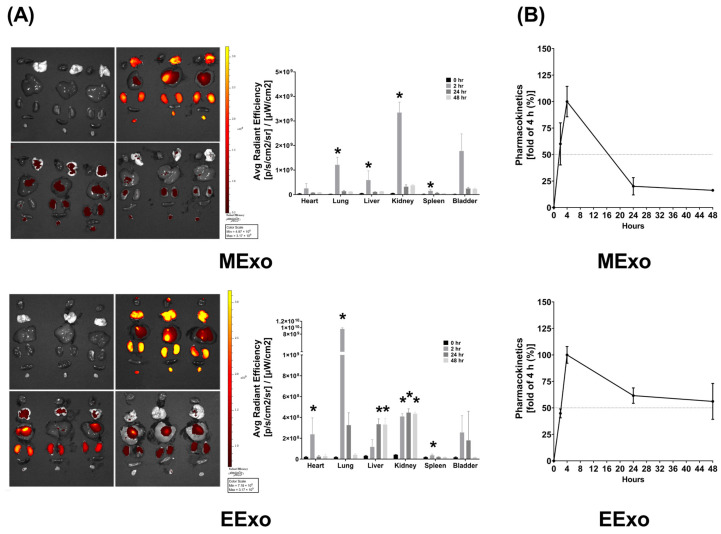
(**A**) Biodistribution of hpMSC exosomes (MExo, 1 × 10^8^ particles per mouse) and engineered exosomes (EExo, 1 × 10^9^ particles per mouse) conjugated with Cy7 mono NHS ester in the heart, lungs, liver, kidney, spleen, and bladder of mice, using ex vivo bioluminescence imaging assay, measured at 0, 2, 24, and 48 h after intraperitoneal administration. Data were obtained from 3 mice sacrificed at each time point in both groups. * *p* < 0.05, versus the 0-h mark. (**B**) Pharmacokinetic analysis of MExo and EExo. The plasma concentrations of MExo and EExo were measured through the assay of Cy7 mono NHS ester signal intensities. The MExo and EExo concentrations were measured at 0.5 h after intraperitoneal administration and were used as the baseline. Data were obtained from 3 mice in each group. hpMSC exosomes: exosomes from human placenta-derived mesenchymal stem cells. Engineered exosomes: engineered exosomes from RAW264.7 cells overexpressing hsa-let-7i-5p. The dashed line indicates a 50% fold change compared to the 4-h mark.

As described in Section 2.6.1, the biodistribution data were obtained from three mice sacrificed at each time point in both groups. In mice receiving Cy7-conjugated hpMSC exosomes, bioluminescence imaging revealed significantly higher Cy7 signal intensities in the lungs, liver, kidney, and spleen at 2 h post-administration compared to baseline (all *p* < 0.05) (Figure 2A). However, at 24 and 48 h post-administration, Cy7 signal intensities in these organs were not significantly different from the baseline (Figure 2A). Interestingly, there were no significant differences in Cy7 signal intensities in the heart and bladder at any time point post-administration (Figure 2A). These findings demonstrate significant biodistribution of hpMSC exosomes in the lungs, liver, kidney, and spleen shortly after administration, with a duration of less than 24 h.

In mice receiving Cy7-conjugated engineered exosomes, bioluminescence imaging showed significantly higher Cy7 signal intensities in the heart, lungs, kidney, and spleen at 2 h post-administration compared to baseline (all *p* < 0.05) (Figure 2A). However, at 24 and 48 h, Cy7 signal intensities in the heart, lungs, and spleen were not significantly different from baseline, while those in the liver and kidney remained significantly elevated (all *p* < 0.05) (Figure 2A). Similar to hpMSC exosomes, there were no significant differences in Cy7 signal intensities in the bladder at any time point post-administration (Figure 2A). These data indicate significant biodistribution of engineered exosomes in the heart, lungs, liver, kidney, and spleen after administration.

The pharmacokinetics data, as described in Section 2.6.2, were obtained by analyzing the serially harvested plasma samples from three mice in each group. Pharmacokinetic analyses revealed that plasma concentrations of both hpMSC and engineered exosomes peaked at 4 h post-administration, with an approximate half-life of 16 h for hpMSC exosomes and 48 h for engineered exosomes (Figure 2B).

### 3.3. Survivorship and Plasma Cytokines

**Figure 3:**
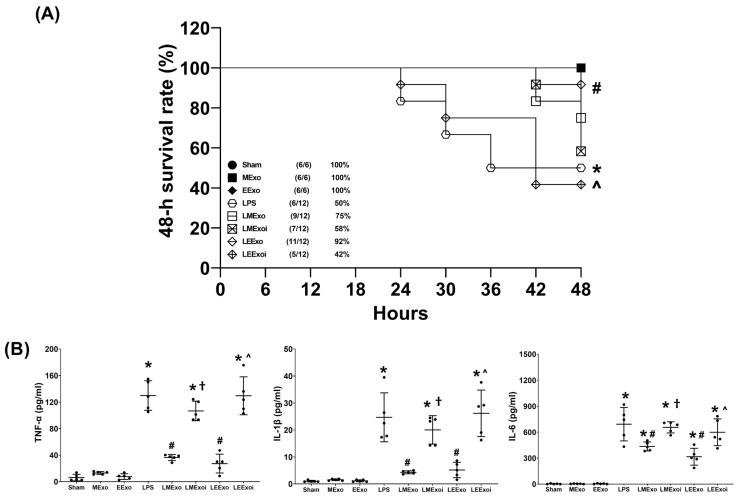
(**A**) The 48-h survival rates, as determined by calculating the number of mice that survived the 48 h observational duration in each group after normal saline or lipopolysaccharide administration. Data were derived from 6 mice in the Sham, MExo, and EExo groups and 12 mice from the LPS, LMExo, LMExoi, LEEXo, and LEExoi groups. * *p* < 0.05, the LPS group versus the Sham group. # *p* < 0.05, the LEExo group versus LPS group. ˄ *p* < 0.05, the LEExoi group versus the LEExo group. (**B**) Plasma concentrations of tumor necrosis factor-α (TNF-α), interleukin-1β (IL-1β), and IL-6, measured using enzyme-linked immunosorbent assay. Data were obtained from 5 mice in each group. All assays were measured at 48 h after normal saline or lipopolysaccharide administration. Data represented as mean ± standard deviations. * *p* < 0.05, versus the Sham group. # *p* < 0.05, versus the LPS group. † *p* < 0.05, the LMExoi group versus the LMExo group. ˄ *p* < 0.05, the LEExoi group versus the LEExo group. Sham: the normal saline group. MExo group: the normal saline plus hpMSC exosome group. EExo group: the normal saline plus engineered exosome group. LPS: the lipopolysaccharide (LPS) group. LMExo: the LPS plus hpMSC exosome group. LMExoi: the LPS plus inhibitor-treated hpMSC exosome group. LEExo: the LPS plus engineered exosome group. LEExoi: the LPS plus inhibitor-treated engineered exosome group.

The 48-h survival rate data, as described in Section 2.7.3, were obtained from an independent cohort of 78 mice, where 18 of them were allocated to the Sham, MExo, and EExo groups (*n* = 6 in each group) and 60 of them were allocated to the LPS, LMExo, LMexoi, LEExo, and LEExoi groups (*n* = 12 in each group). Figure 3A depicts the 48-h survival rates. The 48-h survival rates of the Sham, MExo, and EExo groups all reached 100%. In contrast, the LPS group exhibited a significantly lower 48-h survival rate compared to the Sham group (50% versus 100%, *p* = 0.016), indicating substantial mortality induced by lipopolysaccharide. Notably, the 48-h survival rate in the LEExo group was significantly higher than that in the LPS group (92% versus 50%, *p* = 0.020), demonstrating the ability of engineered exosomes to mitigate lipopolysaccharide’s adverse effects and enhance survivorship in mice. Conversely, the 48-h survival rate in the LEEXoi group was significantly lower than that in the LEExo group (42% versus 92%, *p* = 0.008), indicating that inhibition of hsa-let-7i-5p significantly diminishes the therapeutic effects of engineered exosomes. A similar trend was observed among the LPS, LMExo, and LMExoi groups, although the differences among these groups did not reach statistical significance (all *p* > 0.05). Moreover, the difference between the LMExo and LEExo groups also did not reach statistical significance (75% versus 92%, *p* > 0.05).

The plasma cytokine data presented in Figure 3B were obtained through analyzing the plasma samples harvested from five surviving mice from each group, as described in Section 2.7.3. TNF-α concentrations in the Sham, MExo, and EExo groups were low and significantly elevated in the LPS group compared to the Sham group (*p* < 0.001). The TNF-α concentrations in the LMExo and LEExo groups were compatible, and both groups showed significantly lower TNF-α concentrations compared to the LPS group (both *p* < 0.001). Conversely, TNF-α concentrations were significantly higher in LMExoi and LEExoi groups compared to their respective exosome-treated groups (both *p* < 0.001). Similar patterns were observed for IL-1β and IL-6 data. Overall, these results demonstrate that hpMSC exosomes and engineered exosomes can achieve similar effects on mitigating lipopolysaccharide-induced systemic inflammation. Moreover, inhibition of hsa-let-7i-5p can attenuate the therapeutic effects of both exosome types in this regard.

### 3.4. Lung Injury

**Figure 4:**
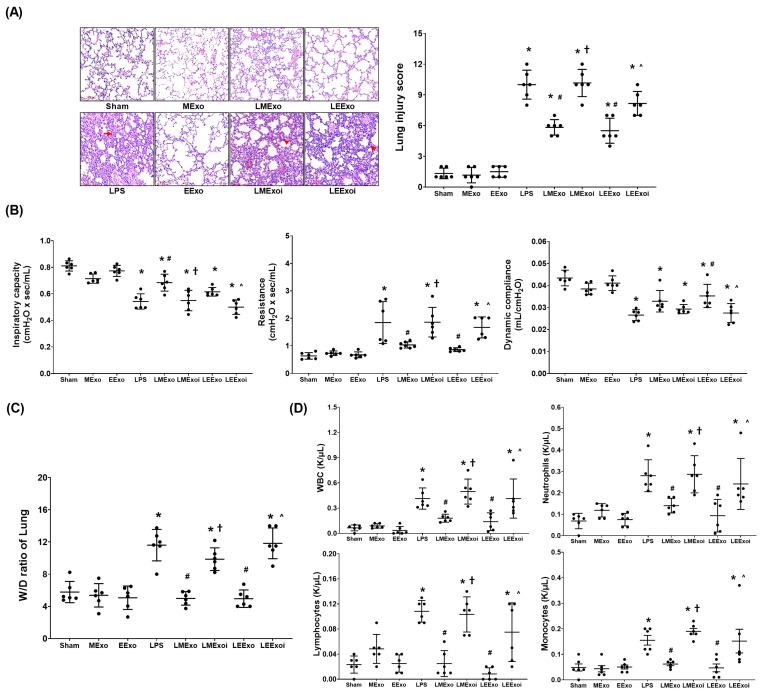
(**A**) Representative histological characteristics of lung injury in lung tissues stained with hematoxylin–eosin, evaluated using a light microscope (200×), and the data of lung injury scores. Data were obtained from 6 mice in each group. (**B**) Lung function, representing inspiratory capacity, airway resistance, and dynamic compliance. Data were obtained from 6 mice from each group for each parameter. (**C**) Wet/dry weight ratio (W/D ratio) of the lung tissues. Data were obtained from 6 mice in each group. (**D**) The cell number of white blood cells (WBCs), neutrophils, lymphocytes, and monocytes in collected bronchoalveolar lavage fluid (BALF). Data were obtained from 6 mice in each group. All assays were measured at 48 h after normal saline or lipopolysaccharide administration. Data represented as mean ± standard deviations. * *p* < 0.05, versus the Sham group. # *p* < 0.05, versus the LPS group. † *p* < 0.05, the LMExoi group versus the LMExo group. ˄ *p* < 0.05, the LEExoi group versus the LEExo group. Sham: the normal saline group. MExo group: the normal saline plus hpMSC exosome group. EExo group: the normal saline plus engineered exosome group. LPS: the lipopolysaccharide (LPS) group. LMExo: the LPS plus hpMSC exosome group. LMExoi: the LPS plus inhibitor-treated hpMSC exosome group. LEExo: the LPS plus engineered exosome group. LEExoi: the LPS plus inhibitor-treated engineered exosome group.

The lung injury data were obtained through analyzing the formaldehyde-infused right lung tissues from six mice in each group, as described in Section 2.7.5 and Section 2.7.6. Figure 4A presents the histological analysis of lung tissues and the corresponding lung injury scores. Histological examination revealed normal lung tissue characteristics in the Sham, MExo, and EExo groups. In contrast, lung tissues from the LPS, LMExo, LMExoi, LEExo, and LEExoi groups exhibited features of lung injury, including increased polymorphonuclear (PMN) infiltration, focal necrosis, and hemorrhages/congestion. Lung injury scores were low in the Sham, MExo, and EExo groups and significantly increased in the LPS group compared to the Sham group (*p* < 0.001). Moreover, lung injury scores in the LMExo and LEExo groups were comparable, and both were significantly reduced compared to the LPS group (both *p* < 0.001). Notably, lung injury scores were significantly higher in the LMExoi and LEExoi groups compared to their respective exosome-treated groups (*p* < 0.001 and *p* = 0.001, respectively).

The lung function assay data presented in Figure 4B were obtained through analyzing the parameters of lung function measured in six mice from each group, as described in Section 2.7.4. Inspiratory capacity and dynamic compliance were significantly lower in the LPS group compared to the Sham group (both *p* < 0.001), while airway resistance was significantly higher in the LPS group (*p* < 0.001). In the LMExo group, inspiratory capacity was significantly higher (*p* = 0.001), and airway resistance was significantly lower (*p* = 0.005) compared to the LPS group. However, dynamic compliance did not significantly differ between the LMExo and LPS groups. Similarly, the LEExo group showed significantly lower airway resistance (*p* = 0.001) and higher dynamic compliance (*p* = 0.005) compared to the LPS group, with no significant difference in inspiratory capacity. Comparing the LMExoi and LMExo groups, inspiratory capacity was significantly lower (*p* = 0.002), and resistance was significantly higher (*p* = 0.004) in the LMExoi group. Similarly, in the LEExoi group compared to the LEExo group, inspiratory capacity and dynamic compliance were significantly lower (*p* = 0.010 and *p* = 0.018, respectively), and resistance was significantly higher (*p* = 0.008). Moreover, the differences in inspiratory capacity, dynamic compliance, and airway resistance between the LMEXo and LEExo groups did not reach statistical significance (all *p* > 0.05).

The wet/dry weight ratio data presented in Figure 4C were obtained through analyzing the lung tissue samples in six mice from each group, as described in Section 2.7.5 and Section 2.7.6. The BALF data presented in Figure 4D were obtained through analyzing the BALF samples from six mice from each group, as described in Section 2.7.5. The data of wet/dry weight ratios of lung tissues (Figure 4C) and the data of cell counts in BALF (Figure 4D) both reflect the trends observed in lung injury scores (Figure 4A). These findings collectively underscore the comparable therapeutic efficacy of hpMSC exosomes and engineered exosomes in attenuating lipopolysaccharide-induced lung injury in mice. Additionally, inhibition of hsa-let-7i-5p diminishes the therapeutic effects of both exosome types in this context.

### 3.5. Lung Inflammation

**Figure 5:**
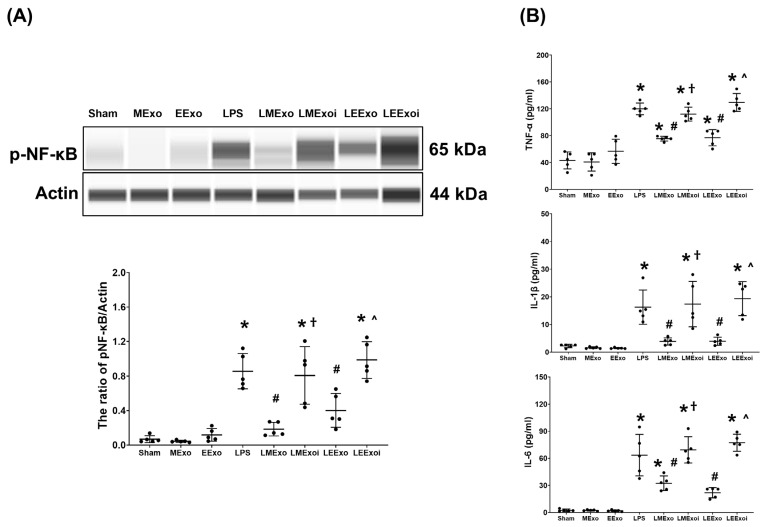
(**A**) Representative gel photography of phosphorylated nuclear factor-kB (p-NF-kB) and Actin (internal standard), assayed via the Simple Western method and the relative band density of p-NF-kB/Actin ratio in lung tissues. Data were obtained from 5 mice in each group. (**B**) The concentrations of tumor necrosis factor-α (TNF-α), interleukin 1-β (IL-1β), and IL-6 in lung tissues, measured via the enzyme-linked immunosorbent assay. Data were obtained from 5 mice in each group. All assays were measured at 48 h after normal saline or lipopolysaccharide administration. Data represented as mean ± standard deviations. * *p* < 0.05, versus the Sham group. # *p* < 0.05, versus the LPS group. † *p* < 0.05, the LMExoi group versus the LMExo group. ˄ *p* < 0.05, the LEExoi group versus the LEExo group. Sham: the normal saline group. MExo group: the normal saline plus hpMSC exosome group. EExo group: the normal saline plus engineered exosome group. LPS: the lipopolysaccharide (LPS) group. LMExo: the LPS plus hpMSC exosome group. LMExoi: the LPS plus inhibitor-treated hpMSC exosome group. LEExo: the LPS plus engineered exosome group. LEExoi: the LPS plus inhibitor-treated engineered exosome group.

Figure 5A illustrates the expression levels of the upstream regulator factor NF-κB in lung tissues. As described in Section 2.7.7, a, the snap-frozen lung tissues from five mice, randomly selected from the six surviving mice in each group, were used for this assay. Phosphorylated-NF-κB (p-NF-κB) expression levels in the Sham, MExo, and EExo groups were minimal. Conversely, lipopolysaccharide significantly upregulated NF-κB, as evidenced by the significantly higher p-NF-κB expression level in the LPS group compared to the Sham group (*p* < 0.001). Notably, expression levels of p-NF-κB in the LMExo and LEExo groups were comparable, and both groups exhibited significantly lower p-NF-κB expression levels compared to the LPS group (*p* < 0.001 and *p* = 0.006, respectively). Moreover, p-NF-κB expression levels were significantly higher in both LMExoi and LEExoi groups compared to their respective exosome-treated groups (both *p* < 0.001).

Also as described in Section 2.7.7, b, the snap-frozen lung tissues from five mice, randomly selected from the six surviving mice in each group, were used for the analysis of cytokines in lung tissues. Figure 5B displays data on TNF-α, IL-1β, and IL-6 expression levels in lung tissues, which mirrored the trends observed in p-NF-κB expression (Figure 5A). These findings collectively underscore the comparable efficacy of hpMSC exosomes and engineered exosomes in attenuating lipopolysaccharide-induced lung inflammation in mice. Furthermore, they provide clear evidence of the pivotal role of hsa-let-7i-5p in mediating the therapeutic effects of hpMSC exosomes and engineered exosomes in this context.

### 3.6. Macrophage Polarization in Lung Tissues

**Figure 6:**
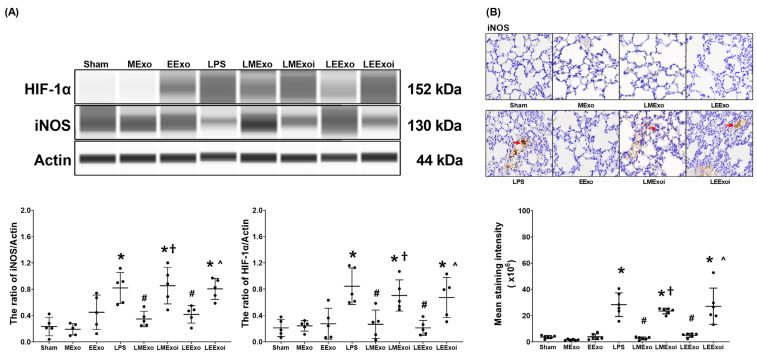
(**A**) Representative gel photography of hypoxia-inducible factor-1α (HIF-1α), inducible nitric oxide synthase (iNOS), and Actin (internal standard), assayed via the Simple Western method and the relative band density of HIF-1α/Actin and iNOS/Actin ratios in lung tissues. Data were obtained from 5 mice in each group. (**B**) Representative microscopy images of immunohistochemistry staining assay of iNOS (marked by the red arrow) and the iNOS quantitative sum intensities in lung tissues. Data were obtained from 5 mice in each group. All assays were measured at 48 h after normal saline or lipopolysaccharide administration. Data represented as mean ± standard deviations. * *p* < 0.05, versus the Sham group. # *p* < 0.05, versus the LPS group. † *p* < 0.05, the LMExoi group versus the LMExo group. ˄ *p* < 0.05, the LEExoi group versus the LEExo group. Sham: the normal saline group. MExo group: the normal saline plus hpMSC exosome group. EExo group: the normal saline plus engineered exosome group. LPS: the lipopolysaccharide (LPS) group. LMExo: the LPS plus hpMSC exosome group. LMExoi: the LPS plus inhibitor-treated hpMSC exosome group. LEExo: the LPS plus engineered exosome group. LEExoi: the LPS plus inhibitor-treated engineered exosome group.

As described in Section 2.7.7, c, the snap-frozen lung tissues from five mice, randomly selected from the six surviving mice in each group, were used for this assay. Figure 6A depicts the expression levels of HIF-1α, the promoter of M1 phase polarization, in lung tissues. Minimal HIF-1α expression was observed in the Sham, MExo, and EExo groups. In contrast, lipopolysaccharide significantly increased HIF-1α expression in the LPS group compared to the Sham group (*p* = 0.001), confirming its effect on upregulating HIF-1α in mouse lung tissues. Importantly, HIF-1α expression levels in the LMExo and LEExo groups were comparable, and both groups exhibited significantly lower HIF-1α expression levels compared to the LPS group (*p* = 0.003 and *p* = 0.001, respectively). Notably, the expression levels of HIF-1α were also significantly higher in the LMExoi and LEExoi groups compared to their respective exosome-treated groups (*p* = 0.049 and *p* = 0.032, respectively).

Expression data of the macrophage M1 phase polarization marker iNOS, presented in Figure 6A, were obtained from the snap-frozen lung tissues from five mice, randomly selected from the six surviving mice in each group, as described in Section 2.7.7, c. The iNOS expression data presented in Figure 6B were obtained from the formaldehyde-infused left lung tissues from five mice, randomly selected from the six surviving mice in each group, as described in Section 2.7.7, c. Data on the expression levels of iNOS in lung tissues (Figure 6A,B) mirrored the trends observed in HIF-1α expression (Figure 6A). These findings collectively highlight the comparable efficacy of hpMSC exosomes and engineered exosomes in attenuating lipopolysaccharide-induced macrophage polarization in mouse lung tissues. Furthermore, they underscore the pivotal role of hsa-let-7i-5p in mediating the therapeutic effects of hpMSC exosomes and engineered exosomes in this context.

### 3.7. Lung Oxidation

**Figure 7:**
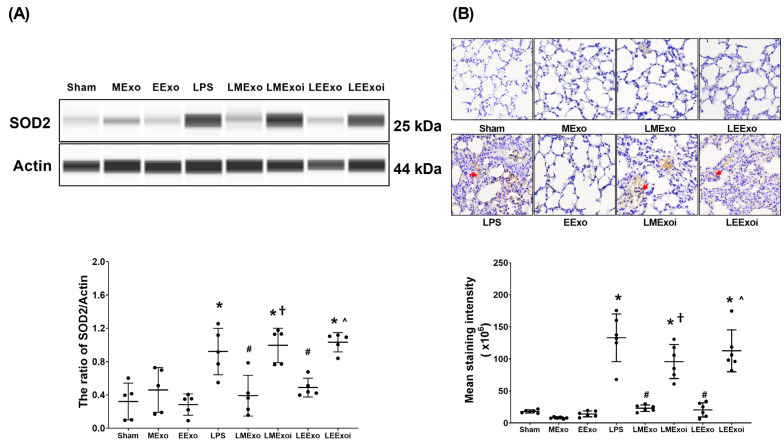
(**A**) Representative gel photography of superoxide dismutase 2 (SOD2) and Actin (internal standard), assayed via the Simple Western method and the relative band density of SOD2/Actin ratio in lung tissues. Data were obtained from 5 mice in each group. (**B**) Representative microscopy images of immunohistochemistry staining assay of myeloperoxidase (MPO, marked by the red arrow) and the MPO quantitative sum intensities in lung tissues. Data were obtained from 5 mice in each group. All assays were measured at 48 h after normal saline or lipopolysaccharide administration. Data represented as mean ± standard deviations. * *p* < 0.05, versus the Sham group. # *p* < 0.05, versus the LPS group. † *p* < 0.05, the LMExoi group versus the LMExo group. ˄ *p* < 0.05, the LEExoi group versus the LEExo group. Sham: the normal saline group. MExo group: the normal saline plus hpMSC exosome group. EExo group: the normal saline plus engineered exosome group. LPS: the lipopolysaccharide (LPS) group. LMExo: the LPS plus hpMSC exosome group. LMExoi: the LPS plus inhibitor-treated hpMSC exosome group. LEExo: the LPS plus engineered exosome group. LEExoi: the LPS plus inhibitor-treated engineered exosome group.

As described in Section 2.7.8, a, the snap-frozen lung tissues from five mice, randomly selected from the six surviving mice in each group, were used for this assay. Figure 7A displays the expression levels of SOD2 in lung tissues. Minimal SOD2 expression was observed in the Sham, MExo, and EExo groups. In contrast, the LPS group exhibited significantly higher SOD2 expression compared to the Sham group (*p* = 0.015). Importantly, SOD2 expression levels in the LMExo and LEExo groups were comparable, and both groups showed significantly lower SOD2 expression levels compared to the LPS group (*p* = 0.007 and =0.043, respectively). Moreover, SOD2 expression was significantly higher in the LMExoi group compared to the LMExo group (*p* = 0.001), and similarly, the LEExoi group exhibited higher SOD2 expression than the LEExo group (*p* = 0.005).

As described in Section 2.7.8, b, the formaldehyde-infused left lung tissues from five mice, randomly selected from the six surviving mice in each group, were used for this assay. In Figure 7B, the lipid peroxidation status in lung tissues is illustrated by the levels of MDA (a lipid peroxidation marker). Low MDA levels were observed in the Sham, MExo, and EExo groups. However, the LPS group showed significantly higher MDA levels compared to the Sham group (*p* < 0.001). Notably, MDA levels in the LMExo and LEExo groups were comparable, and both groups exhibited significantly lower MDA levels compared to the LPS group (both *p* < 0.001). Conversely, MDA levels were significantly higher in the LMExoi group compared to the LMExo group (*p* < 0.001), and similarly, the LEExoi group showed higher MDA levels than the LEExo group (*p* < 0.001).

These findings collectively demonstrate the comparable effectiveness of hpMSC exosomes and engineered exosomes in mitigating lung oxidation induced by lipopolysaccharide in mice. Additionally, they underscore the crucial role of hsa-let-7i-5p in mediating the therapeutic effects of both exosome types in this context.

### 3.8. Lung Apoptosis

**Figure 8:**
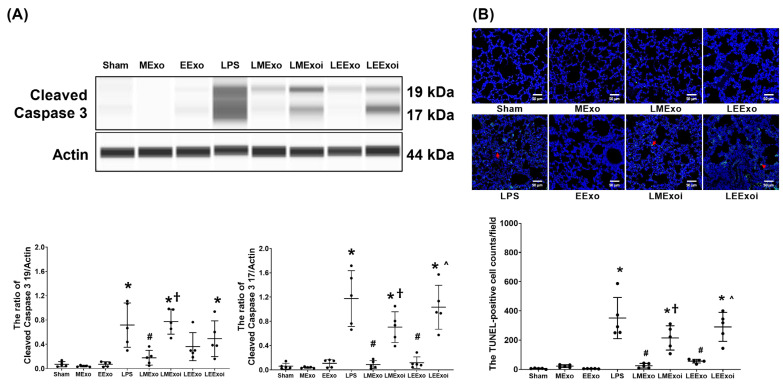
(**A**) Representative gel photography of pro-apoptotic cleaved caspase 3 (19 KDa and 17 KDa) and Actin (internal standard), assayed via the Simple Western method and the relative band density of cleaved caspase 3 (19 KDa)/Actin and cleaved caspase 3 (17 KDa)/Actin ratios in lung tissues. Data were obtained from 5 mice in each group. (**B**) Representative microscopy fluorescent images of TUNEL assay of fragmented DNA (marked by the red arrow) and the TUNEL-positive cell counts in lung tissues. Data were obtained from 5 mice in each group. All assays were measured at 48 h after normal saline or lipopolysaccharide administration. Data represented as mean ± standard deviations. * *p* < 0.05, versus the Sham group. # *p* < 0.05, versus the LPS group. † *p* < 0.05, the LMExoi group versus the LMExo group. ˄ *p* < 0.05, the LEExoi group versus the LEExo group. Sham: the normal saline group. MExo group: the normal saline plus hpMSC exosome group. EExo group: the normal saline plus engineered exosome group. LPS: the lipopolysaccharide (LPS) group. LMExo: the LPS plus hpMSC exosome group. LMExoi: the LPS plus inhibitor-treated hpMSC exosome group. LEExo: the LPS plus engineered exosome group. LEExoi: the LPS plus inhibitor-treated engineered exosome group.

As described in Section 2.7.9, a, the snap-frozen lung tissues from five mice, randomly selected from the six surviving mice in each group, were used for this assay. Figure 8A illustrates the expression levels of the pro-apoptotic protein cleaved caspase-3 (17 KDa and 19 KDa) in lung tissues. Minimal expression of cleaved caspase-3 (17 KDa) was observed in the Sham, MExo, and EExo groups. Conversely, the LPS group exhibited significantly higher expression levels of cleaved caspase-3 (17 KDa) compared to the Sham group (*p* < 0.001). Notably, expression levels of cleaved caspase-3 (17 KDa) in the LMExo and LEExo groups were comparable, and both groups showed significantly lower cleaved caspase-3 (17 KDa) expression levels compared to the LPS group (both *p* < 0.001). In contrast, the expression levels of cleaved caspase-3 (17 KDa) were also significantly higher in the LMExoi and LEExoi groups compared to their respective exosome-treated groups (*p* = 0.004 and *p* < 0.001, respectively). Data of cleaved caspase-3 (19 KDa) were similar to those of cleaved caspase-3 (17 KDa), except that the differences between the LEExo and LPS groups, as well as between the LEExoi and LEExo groups, did not reach statistical significance.

As described in Section 2.7.9, b, the formaldehyde-infused left lung tissues from five mice, randomly selected from the six surviving mice in each group, were used for this assay. In Figure 8B, the apoptosis status in lung tissues is illustrated by data on DNA fragmentation measured using the TUNEL assay and the TUNEL-positive cell count. These results mirrored the trends observed in cleaved caspase-3 (17 KDa) expression (Figure 8A).

These findings collectively demonstrate the comparable effectiveness of hpMSC exosomes and engineered exosomes in mitigating lung apoptosis induced by lipopolysaccharide in mice. Additionally, they underscore the crucial role of hsa-let-7i-5p in mediating the therapeutic effects of both exosome types in this context.

## 4. Discussion

The results of this murine study demonstrate that engineered exosomes enriched with hsa-let-7i-5p, derived from genetically modified RAW264.7 cells overexpressing hsa-let-7i-5p, exhibit the capability to improve survival rates and alleviate systemic inflammation and lung injury in mice subjected to endotoxin treatment. Furthermore, our findings demonstrate that these engineered exosomes can attenuate oxidative stress, inflammation, macrophage polarization, and apoptosis in lung tissues of endotoxin-treated mice. These outcomes lend credence to our hypothesis and underscore the therapeutic promise of engineered exosomes enriched with hsa-let-7i-5p in the treatment of sepsis. Considering the current absence of effective therapies for sepsis [3], the implications of our study are substantial for clinical application.

Cumulative evidence underscores the therapeutic potential of MSC exosome therapy in combating sepsis. For instance, exosomes derived from bone marrow MSCs have been shown to alleviate acute lung injury in septic mice treated with endotoxin [28]. Our prior research also provides compelling evidence of the therapeutic benefits of exosomes derived from the placental choriodecidual membrane MSCs (specifically, hpMSC exosomes studied herein) in enhancing survival rates and ameliorating acute lung and liver injuries in obese mice with endotoxemia [18,21]. Consistent findings can be observed in the current investigation. Our results reveal that hpMSC exosomes enhance the 48-h survival rate and alleviate acute lung injury in endotoxin-treated mice, with mechanisms potentially involving the inhibition of systemic and pulmonary inflammation, as well as oxidation and apoptosis in lung tissues. The hpMSC exosome data from this study, along with previous findings [18,21,28], advocate for the incorporation of MSC exosomes into sepsis treatment as a promising therapeutic approach. Given the ongoing scarcity of effective therapies for sepsis [3], the clinical significance of these findings is substantial.

However, as previously mentioned, one of the primary challenges hindering the clinical application of MSC exosomes is the limited availability of MSC sources [23]. Moreover, ethical and political controversies surrounding stem cell therapy have been raised [29]. It would be advantageous if the therapeutic benefits offered by MSC exosomes could be reproduced using exosomes derived from non-stem cell sources. Achieving this objective hinges on identifying the key components within MSC exosomes that mediate their therapeutic effects against sepsis. Our earlier research underscored the pivotal role of hsa-let-7i-5p in this context, revealing it as the most abundant microRNA present in hpMSC exosomes. Additionally, previous findings have highlighted the anti-inflammatory and antioxidative properties of hsa-let-7i-5p [22]. Further supporting this notion, our previous studies demonstrated that inhibiting this microRNA can nullify the therapeutic effects of hpMSC exosomes against sepsis in obese mice [18,21]. Building upon these insights, we employed a plasmid-mediated genetic modification of RAW264.7 cells to produce engineered exosomes enriched with hsa-let-7i-5p.

Our analysis confirms that the engineered exosomes are rich in hsa-let-7i-5p, with the miRNA concentration of hsa-let-7i-5p in engineered exosomes from genetically modified RAW264.7 cells being approximately 16-fold higher than that in exosomes from naive RAW264.7 cells (Figure 1D). Furthermore, our data demonstrate that the miRNA concentrations of hsa-let-7i-5p in engineered exosomes are comparable to those in hpMSC exosomes (Figure 1). To determine the effective dosage of engineered exosomes against endotoxin-induced sepsis, we conducted a series of preliminary studies. The data from the preliminary studies revealed that engineered exosomes at a dosage of 1 × 10^9^ particles per mouse, but not 1 x 10^8^ particles per mouse (the effective dosage of hpMSC exosomes against sepsis in obese mice, as previously reported [18,21]), significantly improve survivorship (please refer to the Supplemental Figure S1). Consequently, this study opted to utilize this dosage of engineered exosomes (ten times higher than that of hpMSC exosomes) for further investigation. Following intraperitoneal administration, we observed significant biodistribution of engineered exosomes in major organs, particularly in the lungs, liver, and kidneys, akin to the distribution of hpMSC exosomes (Figure 2). The in vivo half-life of engineered exosomes is approximately 48 h after intraperitoneal administration, compared to the shorter half-life of hpMSC exosomes (approximately 16 h after intraperitoneal administration) (Figure 2). Based on these findings, we designed and conducted the present study to compare the therapeutic effects of engineered exosomes (at the dosage of 1 × 10^9^ particles per mouse) and hpMSC exosomes (1 × 10^8^ particles per mouse) against endotoxin-induced sepsis in mice. As summarized in previous sections, our data clearly demonstrate that engineered exosomes can improve the 48-h survivor rate and alleviate acute lung injury in septic mice with endotoxemia.

Our data further demonstrate that the therapeutic effects of engineered exosomes against sepsis are similar to those of hpMSC exosomes. These data thus provide clear evidence to support the concept that engineered exosomes from non-stem cell sources (for example, RAW264.7 cells) can serve as a satisfactory alternative to MSC exosomes in the treatment of sepsis. Moreover, data from the present study further demonstrate the common mechanisms of anti-inflammation, anti-oxidation, and inhibiting the cell death process of apoptosis in underscoring the observed therapeutic effects of engineered exosomes and hpMSC exosomes. These data are in line with previous data regarding the anti-inflammation and anti-oxidation effects of hsa-let-7i-5p [22]. Moreover, data from the present study show that inhibiting hsa-let-7i-5p can abrogate the therapeutic effects of both engineered exosomes and hpMSC exosomes against sepsis. Collectively, these data highlight the crucial role of hsa-let-7i-5p in mediating the therapeutic effects of engineered exosomes enriched in hsa-let-7i-5p as well as hpMSC exosomes against sepsis.

Data from the present study highlight that hsa-let-7i-5p can inhibit endotoxin-induced activation of NF-κB and HIF-1α, the upstream regulators of inflammation and macrophage activation, in lung tissues of septic mice. These findings suggest that hsa-let-7i-5p likely exerts its anti-inflammatory effects and modulates cytokine upregulation and macrophage activation by inhibiting NF-κB and HIF-1α activation. Additionally, oxidative stress and inflammation may collaboratively contribute to mitochondrial injury and dysfunction [30], which in turn may synergistically induce apoptosis [31]. Given hsa-let-7i-5p’s ability to mitigate inflammation and oxidation, it is reasonable to observe that engineered exosomes enriched in hsa-let-7i-5p can mitigate apoptosis in lung tissue of septic mice with endotoxemia. However, the exact mechanism underlying the effects of hsa-let-7i-5p in inhibiting NF-κB and HIF-1α activation remains to be elucidated. It is known that endotoxin-induced expression of NF-κB and HIF-1α is regulated by toll-like receptor 4 (TLR4) [32,33]. Therefore, we speculate that hsa-let-7i-5p may act by inhibiting TLR4 activation to exert its effects in this context. This hypothesis is supported by predictive data from a curated database, MiRTarBase, suggesting that TLR4 is one of the downstream targets of hsa-let-7i-5p. This prediction implies that hsa-let-7i-5p may function by binding to the 3′-untranslated region of TLR4 mRNA and consequently reducing the translation of TLR4 at the post-transcriptional level. To further elucidate this issue, a follow-up study is currently underway in our laboratory.

Of note, certain limitations exist within the study. Firstly, the mechanism underlying our findings reveals that a dosage ten times higher is required for engineered exosomes enriched in hsa-let-7i-5p to achieve therapeutic effects comparable to those of hpMSC exosomes. One possible explanation for this discrepancy may be attributed to the variance in therapeutic elements, specifically miRNA content, between engineered exosomes and hpMSC exosomes. While our engineered exosomes primarily contain hsa-let-7i-5p due to the genetic modification of RAW264.7 cells, hpMSC exosomes contain a broader array of miRNAs, including hsa-miR-612, as previously demonstrated in our data. According to predictive data from MiRTarBase, hsa-miR-612 targets TFB1M, a mitochondrial methyltransferase crucial in regulating mitochondrial DNA transcription [34]. Given the significance of mitochondrial dysfunction in sepsis, it is plausible that hsa-miR-612 delivered by hpMSC exosomes may modulate sepsis-induced mitochondrial dysfunction via TFB1M regulation, thereby contributing to the observed therapeutic effects. This suggests that incorporating additional miRNAs with therapeutic potential into engineered exosomes could enhance their efficacy against sepsis and merits further investigation. Secondly, beyond genetic modification, exosomes enriched in specific miRNA can be achieved through alternative techniques such as electroporation [35]. The optimal technique for generating engineered exosomes with maximal efficiency and therapeutic efficacy against sepsis warrants clarification. Thirdly, this study utilized RAW264.7 cells for engineered exosome production. Given that RAW264.7 cells are a murine macrophage cell line, the transferability of these engineered exosomes’ effects to other species, such as Homo sapiens, remains to be explored. Fourthly, this study utilized MSC exosomes derived from human placenta to facilitate investigations. MSCs are considered to have low immunogenicity [36], a characteristic that allows for the allogeneic and xenogeneic transplantation of MSCs [37]. Data from this study largely support this concept, as we observed no significant difference between the Sham and MExo groups. However, as this study did not directly investigate the immune response to the administration of MSC exosomes from human placenta, more data are needed before further conclusions can be made. Fifthly, our analysis unveiled that both engineered exosomes and MSC exosomes exhibited particle sizes ranging from approximately 50 to 200 nm (Figure 1C). Interestingly, our data demonstrated a more heterogeneous particle size distribution in engineered exosomes compared to MSC exosomes. The mechanisms underlying and the implications of this disparity in particle sizing between engineered exosomes and MSC exosomes are yet to be fully understood. Further research exploring the biological origins and functional consequences of these differences could provide valuable insights into optimizing the design and application of engineered exosomes for various therapeutic purposes. Sixthly, data from this study suggest a longer in vivo half-life of engineered exosomes compared to MSC exosomes (48 versus 16 h) (Figure 1D). However, it is important to note that we administered a dose of engineered exosomes ten times higher than that of MSC exosomes for this measurement, potentially biasing the comparison of the data. Further investigation is necessary in the future before drawing definitive conclusions. Seventhly, our results exhibit some interrelation without adequate diversity. This could be attributed to the focus of our investigation, primarily on inflammation and oxidation mechanisms. To address this limitation, future studies should explore additional crucial mechanisms to ensure a more comprehensive understanding of the topic. Finally, the data trend prominently showcases the impact of engineered exosomes enriched with hsa-let-7i-5p, as well as hpMSC exosomes, in alleviating acute lung injury induced by endotoxemia. Nonetheless, it is crucial to note that the overall sample size is relatively modest, and certain assay results stem from a limited number of mice (for instance, only three mice per group for immunoblotting assays). Additionally, it is important to acknowledge that this study was conducted on murine subjects. Hence, caution should be exercised when interpreting and applying these findings further.

This study presents compelling evidence that engineered exosomes enriched with hsa-let-7i-5p offer therapeutic benefits comparable to hpMSCs in treating sepsis, marking a potential breakthrough. It underscores the significance of specific molecular mechanisms in exosome-based therapies, prompting further investigation into their precise roles. These findings hold promising implications for the development of engineered exosome-based treatments for sepsis, suggesting avenues for optimization and clinical translation. Additionally, the success of engineered exosomes derived from non-stem cell sources, such as RAW264.7 cells, suggests a promising direction for therapies utilizing alternative cell types, potentially expanding treatment options and circumventing limitations associated with stem cell-based approaches. Ultimately, this research paves the way for innovative therapeutic strategies in combating sepsis and related conditions.

## 5. Conclusions

Engineered exosomes, enriched in hsa-let-7i-5p from RAW264.7 cells overexpressing hsa-let-7i-5p, exhibit significant therapeutic efficacy comparable to that of hpMSC exosomes against sepsis in mice with endotoxemia. Therefore, engineered exosomes enriched with hsa-let-7i-5p present a promising alternative to hpMSC exosomes for the treatment of sepsis.

## 6. Patents

Patent pending: The intervention under investigation in this study involves engineered exosomes loaded with hsa-let-7i-5p, which is presently undergoing evaluation under American Patent (No. 63/557,089) held by C.Y.C. and C.J.H.

## Data Availability

All study data can be found within this article. Data can be made available upon request.

## Supplementary Materials

The following supporting information can be downloaded at: https://www.mdpi.com/article/10.3390/jpm14060619/s1.
