# Supplementary figures and images for: Therapeutic Effects of Engineered Exosomes from RAW264.7 Cells Overexpressing hsa-let-7i-5p against Sepsis in Mice—A Comparative Study with Human Placenta-Derived Mesenchymal Stem Cell Exosomes
Source: J Pers Med. 2024 Jun 9;14(6):619. doi: 10.3390/jpm14060619 (PMC11204613; doi:10.3390/jpm14060619)

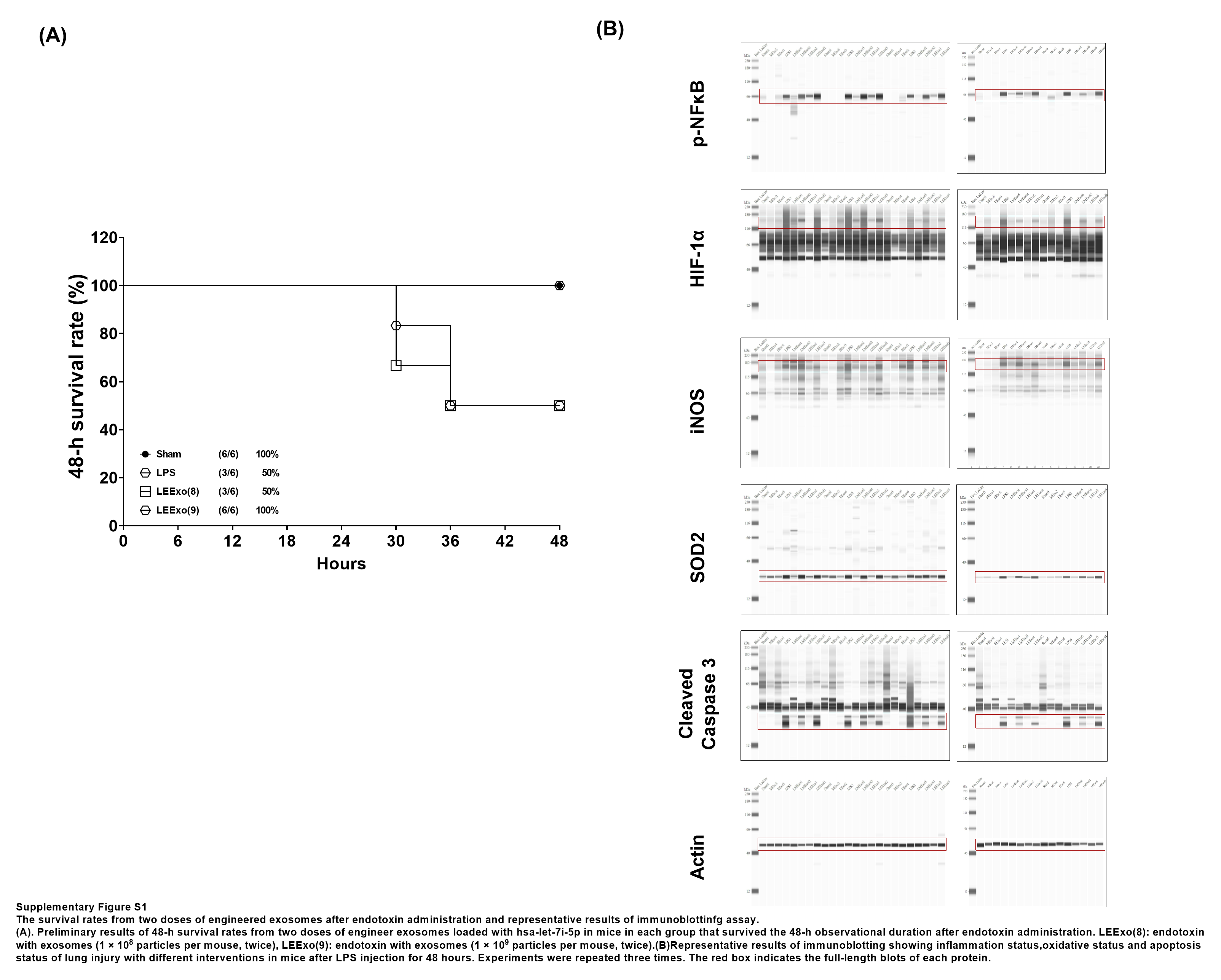

Supplement: Supplementary file 1 [file jpm-14-00619-s001.zip › jpm-2967442-supplementary.tif]
